# Technical efficiency of peripheral health units in Pujehun district of Sierra Leone: a DEA application

**DOI:** 10.1186/1472-6963-5-77

**Published:** 2005-12-14

**Authors:** Ade Renner, Joses M Kirigia, Eyob A Zere, Saidou P Barry, Doris G Kirigia, Clifford Kamara, Lenity HK Muthuri

**Affiliations:** 1 World Health Organization, Country Office, Freetown, Sierra Leone; 2 World Health Organization, Regional Office for Africa, B.P. 06, Brazzaville, Congo; 3 World Health Organization, Country Office, Windhoek, Namibia; 4 University of New South Wales, School of Public Health and community medicine, Australia; 5 Department of Planning and Information, Ministry of Health and Sanitation, Freetown, Sierra Leone; 6 School of Public Health, Department of Health Sciences, Kenyatta University, Nairobi, Kenya

## Abstract

**Background:**

The Data Envelopment Analysis (DEA) method has been fruitfully used in many countries in Asia, Europe and North America to shed light on the efficiency of health facilities and programmes. There is, however, a dearth of such studies in countries in sub-Saharan Africa. Since hospitals and health centres are important instruments in the efforts to scale up pro-poor cost-effective interventions aimed at achieving the United Nations Millennium Development Goals, decision-makers need to ensure that these health facilities provide efficient services. The objective of this study was to measure the technical efficiency (TE) and scale efficiency (SE) of a sample of public peripheral health units (PHUs) in Sierra Leone.

**Methods:**

This study applied the Data Envelopment Analysis approach to investigate the TE and SE among a sample of 37 PHUs in Sierra Leone.

**Results:**

Twenty-two (59%) of the 37 health units analysed were found to be technically inefficient, with an average score of 63% (standard deviation = 18%). On the other hand, 24 (65%) health units were found to be scale inefficient, with an average scale efficiency score of 72% (standard deviation = 17%).

**Conclusion:**

It is concluded that with the existing high levels of pure technical and scale inefficiency, scaling up of interventions to achieve both global and regional targets such as the MDG and Abuja health targets becomes far-fetched. In a country with per capita expenditure on health of about US$7, and with only 30% of its population having access to health services, it is demonstrated that efficiency savings can significantly augment the government's initiatives to cater for the unmet health care needs of the population. Therefore, we strongly recommend that Sierra Leone and all other countries in the Region should institutionalise health facility efficiency monitoring at the Ministry of Health headquarter (MoH/HQ) and at each health district headquarter.

## Background

*"Public health is the science and art of preventing disease, prolonging life and promoting health and **efficiency **through organized community effort." *[1]

Located in West Africa, Sierra Leone has a population of 4.6 million and a total fertility rate of 6.5. Its health indicators are poor. For example, life expectancy at birth is 34.2 years; the probability of dying (per 1000 live births) before the age of 5 years is 313 and between 15 and 59 years is 619 [2]. The number of maternal deaths per 100000 live births is 2000 [3]. These dismal health indicators are a reflection of poor governance [4], poor macroeconomic performance [5] and poor national health system performance [6].

The total expenditure on health as a percentage of the gross domestic product (GDP) increased from 2.6% in 1996 to 4.3% in 2000 [2]. General government expenditure on health constituted 60% of the total expenditure on health; the remaining 40% came from private households and out-of-pocket spending. The fact that health indicators had continued to decline [7] in spite of health expenditure increases could be partly due to an inefficient public health system.

Peripheral health units (PHUs) are a vital part of Sierra Leone's public health system. Given their strategic location in the midst of communities, they constitute an invaluable vehicle for 'organizing community effort for the sanitation of the environment, the control of communicable infections, the education of the individual in personal hygiene and the organization of medical and nursing services for the early diagnosis and preventive treatment of disease' [1]. PHUs are instrumental in efforts to scale up pro-poor package of cost-effective interventions aimed at achieving the U.N. Millennium Development Goals [8,9].

We concur with the father of public health, C.E.A. Winslow [1], that a part of the mandate of the public health discipline ought to be promotion of efficiency, i.e. to maximize the benefit of health interventions (promotion, prevention and preventive treatment) to communities at large from available resources. Therefore, decision-makers need to ensure that PHUs (and all other branches of the public health system) provide services efficiently.

In Sierra Leone no studies of the efficiency of health facilities have been conducted using Data Envelopment Analysis (DEA). This study will therefore be significant in assessing efficiency using more robust techniques and generate information that will be useful for policy, planning and operational management.

The objectives of this study were: (i) to measure the technical and scale efficiency among a sample of public PHUs in Sierra Leone employing the DEA method; and (ii) to demonstrate how its results could be used in the pursuit of the public health objective of promoting efficiency in health facilities.

## Methods

### Overview of Sierra Leone health care delivery system

The Ministry of Health and Sanitation (MOHS) provides about 50% of health care services. The remainder is provided by the private sector (private-for-profit institutions and traditional healers) and national (e.g. Christian Health Association of Sierra Leone) and international (e.g. German Leprosy Rehabilitation Association and *Medecins Sans Frontieres*) NGOs [10].

The country has 13 health districts, each with a District Health Management Team responsible for the implementation, supervision and monitoring of health programmes in the district. Sierra Leone has a total of 31 government hospitals, 22 mission hospitals/clinics, 78 private hospitals/clinics and a network of 788 PHUs. As indicated in Table 1, there are geographical inequities in the distribution of health facilities in the country [10].

**Table 1:** Functioning PHUs and hospitals

**Health District**	**Government PHUs**	**Government Hospitals**	**Mission Hospitals/clinics**	**Private hospitals/clinics**
Bo	87	1	2	3
Kenema	85	2	3	3
Moyamba	95	1	3	5
Port Loko	74	2	2	1
Bombali	66	1	2	9
Kailahun	57	1	2	3
Koinadugu	51	1	0	0
Kono	61	1	2	1
Tonkolili	60	1	2	1
Kambia	35	2	1	1
Pejehun	46	5	0	0
Bonthe	34	1	2	3
Western Area	37	12	1	48
**Total**	788	31	22	78

Source: WHO Regional Office for Africa [10]

Table 2 provides estimates of the number and ratio of human resources for health in 2002. Approximately 63% of the health workers were employed by the government and the remaining by NGOs and private-for-profit institutions.

**Table 2:** Estimated number and ratio of health personnel in 2002

**Categories of Human Resources**	**Number employed by Government**	**Number employed by NGOs & Private sector**	**Total number of human resources**	**Population per health worker**
Doctors	169	131	300	15290
Nurses	406	200	606	7569
Other nursing personnel	1655	1500	3155	1454
Pharmacists	11	-	11	417000
Dispensing technicians	124	-	124	36992
Environmental health officers	168	36	204	22485
Endemic disease control assistant	332	-	332	13816
Community health officers	194	90	284	16151
Laboratory technicians	28	12	40	114675
Radiographers	4	8	12	382250
Sanitary Engineers	2	0	2	2293500
Health education officers	4	1	5	917400
Other health workers	294	-	294	15602

Source: WHO Regional Office for Africa [10]

### Data

Input and output data were analysed for the year 2000. Due to research resource constraints, the planning and information department at the MOHS decided to choose one health district for the study of PHUs. The choice of the study district was done using a simple random sampling technique. This process led to the choice of Pujehun District. Even though there are 46 PHUs in Pujehun today, in the year 2000 there were only 39 PHUs. The data were collected by Pujehun District Health Team using the primary health care facility efficiency analysis data collection instrument of the WHO Regional Office for Africa [11].

### Overview of a public health system

**Figure 1:**
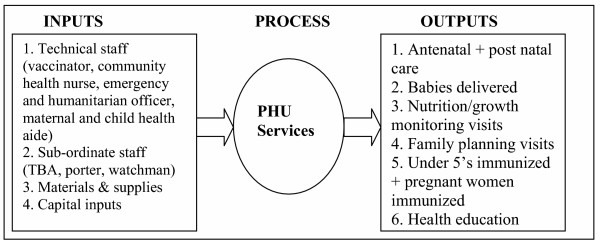
Relationship between inputs and the production process and resulting outputs.

Turnock [12] developed a conceptual framework that ties together the mission and functions of public health to the inputs, processes, outputs and outcomes of the system (see Figure 1). He stated that health systems combine inputs (human, organizational, informational, financial and other resources) to produce outputs (programmes or services or interventions) intended to ultimately yield health or quality-of-life outcomes. In terms of measurability, the author posits that many inputs such as human, financial and organizational resources are easily counted or measured. He further explains that outputs (e.g. number of antenatal care visits, number of immunizations provided, number of people who receive health education and number of condoms distributed) are also generally easy to recognize and count. Following Turnock [12], a public health practice, such as a health centre, employs multiple inputs to produce multiple outputs.

DEA (a non-parametric method) defines efficiency as the ratio of the weighted sum of outputs of a health centre to its weighted sum of inputs [13]. It is particularly useful in public sector organizations (e.g. health facilities) that lack the profit maximization motive and employ a multiple input, multiple output production process. The technical efficiency (TE) of PHUs was found by solving the following linear programming problem for each health unit in the sample:

Max h0=∑r=1suryrj0
 MathType@MTEF@5@5@+=feaafiart1ev1aaatCvAUfKttLearuWrP9MDH5MBPbIqV92AaeXatLxBI9gBaebbnrfifHhDYfgasaacH8akY=wiFfYdH8Gipec8Eeeu0xXdbba9frFj0=OqFfea0dXdd9vqai=hGuQ8kuc9pgc9s8qqaq=dirpe0xb9q8qiLsFr0=vr0=vr0dc8meaabaqaciaacaGaaeqabaqabeGadaaakeaacqWGnbqtcqWGHbqycqWG4baEcaaMc8UaemiAaG2aaSbaaSqaaiabicdaWaqabaGccqGH9aqpdaaeWbqaaiabdwha1naaBaaaleaacqWGYbGCaeqaaOGaemyEaK3aaSbaaSqaaiabdkhaYjabdQgaQnaaBaaameaacqaIWaamaeqaaaWcbeaaaeaacqWGYbGCcqGH9aqpcqaIXaqmaeaacqWGZbWCa0GaeyyeIuoaaaa@4557@

Subject to:

∑i=1mvixij0=1∑r=1suryrj−∑vixij≤0,j=1,…,nur,vi≥0
 MathType@MTEF@5@5@+=feaafiart1ev1aaatCvAUfKttLearuWrP9MDH5MBPbIqV92AaeXatLxBI9gBaebbnrfifHhDYfgasaacH8akY=wiFfYdH8Gipec8Eeeu0xXdbba9frFj0=OqFfea0dXdd9vqai=hGuQ8kuc9pgc9s8qqaq=dirpe0xb9q8qiLsFr0=vr0=vr0dc8meaabaqaciaacaGaaeqabaqabeGadaaakeaafaqaaeWacaaabaWaaabCaeaacqWG2bGDdaWgaaWcbaGaemyAaKgabeaakiabdIha4naaBaaaleaacqWGPbqAcqWGQbGAdaWgaaadbaGaeGimaadabeaaaSqabaaabaGaemyAaKMaeyypa0JaeGymaedabaGaemyBa0ganiabggHiLdGccqGH9aqpcqaIXaqmaeaaaeaadaaeWbqaaiabdwha1naaBaaaleaacqWGYbGCaeqaaOGaemyEaK3aaSbaaSqaaiabdkhaYjabdQgaQbqabaGccqGHsisldaaeabqaaiabdAha2naaBaaaleaacqWGPbqAaeqaaOGaemiEaG3aaSbaaSqaaiabdMgaPjabdQgaQbqabaGccqGHKjYOcqaIWaamcqGGSaalaSqabeqaniabggHiLdaaleaacqWGYbGCcqGH9aqpcqaIXaqmaeaacqWGZbWCa0GaeyyeIuoaaOqaaiabdQgaQjabg2da9iabigdaXiabcYcaSiablAciljabcYcaSiabd6gaUbqaaiabdwha1naaBaaaleaacqWGYbGCaeqaaOGaeiilaWIaemODay3aaSbaaSqaaiabdMgaPbqabaGccqGHLjYScqaIWaamaeaaaaaaaa@6C1C@

Where:

*y*_*rj *_= amount of output *r *from health centre *j*

*x*_*ij *_= amount of input *i *to health centre *j*

*u*_*r *_= weight given to output *r*

*v*_*i *_= weight given to input *i*

*n *= number of hospitals

*s *= number of outputs

*m *= number of inputs

This mathematical programming technique establishes a production possibilities frontier based on relatively efficient health centres and measures how far the inefficient health centres are from this 'best' practice frontier [14]. The efficient health centres lie on the frontier and are assigned a score of 1 or 100%. Inefficient health centres are allocated a score that is less than 1 (or 100%). The higher the score, the greater the efficiency, and vice versa.

### Model specification

The variable returns to scale (VRS) model was estimated to facilitate the estimation of scale efficiency. It assumed that changes in inputs would lead to disproportionate changes in outputs. In other words, a percentage increase in input can yield less than a percentage change in output signifying diseconomies of scale, or more than a percentage increase of output implying existence of economies of scale. The scale efficiency (SE) is the ratio of constant returns to scale technical efficiency (TE_CRS_) to variable returns to scale technical efficiency (TE_VRS_), i.e. SE = (TE_CRS_)/(TE_VRS_) [15]. All the analysis was undertaken using DEAP, the software developed by Coelli [16].

### Output orientation

The output-oriented DEA model was used for the analysis because the management of PHUs had no control over inputs, especially the deployment of human resources. However, given their public health orientation, PHU staff had a duty to induce demand (through health promotion strategies) for preventive health care services such as antenatal care, family planning services, immunizations, etc. Through their outreach public health work among communities, PHU staff were also supposed to mobilize community efforts and other resources to provide clean water and hygienic human waste disposal facilities, e.g. vented improved pit latrines, especially in rural areas and slums.

As one can see in Table 3, there is serious population under-coverage of the various interventions in Sierra Leone. This is mainly due to critical resource constraints, e.g. per capita total expenditure on health is only US$7 compared to the US$34 per person recommended by the WHO Commission for Macroeconomics and Health [8]. This implies that although there is a large unmet need for primary health care among communities, severe budgetary constraints make it difficult to increase inputs, even assuming that PHUs have control over inputs (which they do not have). Even where inputs (e.g. labour) might be under utilized, it is not within their power to dispose of excess inputs. We felt that output maximization is the most appropriate orientation for health centres which are given a fixed input and requested to produce as much output as possible. Thus, an output-oriented approach focused on the amount by which health unit outputs could be expanded with the same level of inputs.

**Table 3:** Manifestations of inaccessibility to basic health services in Sierra Leone

**Health Manifestations**		**Percentage of population without access**
Pregnant women without access to prenatal/antenatal care		32
Pregnant women without access to trained attendants during childbirth		58
Married women aged 15–49 years not using contraceptives		96
Newborns weighing less than 2.5 kg at birth		22
Children (0–59 months) whose weight falls below minus two standard deviations of the median of the international (NCHS) reference population		27.2
Children (0–59 months) whose weight falls below minus three standard deviations of the median of the international (NCHS) reference population		8.7
Children (0–59 months) suffering moderate stunting		33.9
Children (0–59 months) suffering severe stunting		15.8
Children (0–59 months) suffering moderate wasting		9.8
Children (0–59 months) suffering severe wasting		1.9
Infants not fully immunized with	BCG	61
	DPT3	76
	OPV3	74
	Measles	57
	TT2	80
Population without access to safe water		57
Population without access to sanitation facilities		57
Population without access to health care services		70
Per capita total expenditure on health (US$)		7

Sources: UNICEF [19] and WHO/AFRO [20]

Furthermore, the output- and input-oriented models will estimate exactly the same frontier, and therefore, by definition identify the same set of PHUs (firms) as being efficient. It is only the efficiency measures associated with the inefficient firms that may differ between the two methods [16]. In fact under the assumption of constant returns to scale, even the efficiency scores will not change. We, therefore, feel that the choice of model is not going to affect the results significantly.

### DEA inputs and outputs

The DEA model was estimated with a total of eight variables: six outputs and two inputs. The six outputs for each individual PHU were: (i) number of antenatal plus post-natal visits; (ii) number of child deliveries; (iii) nutritional/child growth monitoring visits; (iv) number of family planning visits; (v) number of children under the age of 5 years immunized plus pregnant women immunized with tetanus toxoid (TT); and (vi) total number of health education sessions conducted through home visits, public meetings, school lectures and outpatient department. PHUs in Sierra Leone did not provide curative care; they were dedicated fully to the provision of health promotion and disease prevention services. The two inputs were: (i) technical staff (community health nurse, vaccinator and maternal and child health aide); and (ii) subordinate staff (including traditional birth attendants, porters and watchmen). The choice of inputs and outputs was guided by the public health conceptual framework and past studies.

## Results

Data for two of the sampled PHUs was incomplete, and thus analysis was based on data from 37 health units of Pujehun District. Table 4 presents descriptive statistics for the outputs and inputs of the 37 public PHUs.

**Table 4:** Means and standard deviations for public PHUs outputs and inputs

**Variables**	**Total**	**Mean**	**Standard deviation**	**Maximum**	**Minimum**
***Outputs:*** Antenatal plus postnatal care visits	25 099	678	749	4080	130
Number of deliveries	4 863	131	99	445	14
Nutrition/growth monitoring visits	29 633	801	1 045	4555	0
Family planning visits	2 958	80	55	252	10
Number of children under the age of 5 years and pregnant women fully immunized	33 399	903	846	4422	193
Health education sessions	7 458	202	118	434	55
***Inputs:***Technical staff	78	2	1	5	1
Subordinate staff	27	1	1	3	0

The TE and SE scores for individual PHUs are given in Table 5. Out of the 37 PHUs, 15 (41%) were found to be technically efficient with a TE score of 100%. The remaining 22 (59%) were technically inefficient since they had a TE score of less than 100%. Seven (47%) of the inefficient PHUs had a TE score of less than 50%. The overall sample average TE score was 78% (standard deviation (SD) = 23%). This implies that if the inefficient PHUs were to operate as efficiently as their peers on the efficient frontier, outputs can be increased by about 22% without changing the quantity of inputs used. The average TE score among the inefficient PHUs was 63% (SD = 18%).

**Table 5:** Technical and scale efficiency scores for public PHUs

**DMU (Health unit)**	**Technical efficiency**	**Scale efficiency**	**DMU (Health unit)**	**Technical efficiency**	**Scale efficiency**
Potoru	100	100	S/Malen	77	54
Gbahama	100	100	Karlu	77	48
Jendema	100	100	Futa Peje	72	62
Saama P	100	100	Kpowubu	72	96
Geoma	100	100	Falaba	71	58
Gissiwulo	100	100	Gbaa	70	84
S/Griema	100	100	Sulima	65	85
Sengema	100	100	Bomu Sa	64	58
Massam	100	100	Vaama	60	100
Static Pu	100	93	Pehala	55	93
Gbondapi	100	88	Gofor	55	64
Zimmi	100	86	T/Barri	49	100
Banjadum	100	84	Saahun	49	100
Bandajum	100	75	Fairo	46	60
Taninahu	100	59	Waiima	45	100
B/Massaq	97	62	Bumpeh	45	57
Dandabu	90	44	Kowama	37	80
S/Kpaka	87	99	S/Bessima	26	85
Njaluahu	82	57			

About 65% of the PHUs were found to be scale inefficient, that is, they suffered from inefficiencies emanating from inappropriate size, i.e. being too small or too large. The average SE score for the sampled PHUs was 82%. This implies that if all PHUs had an optimal size, output would have increased by about 22% without increasing the input consumption. The scale inefficient PHUs had an average SE score of 72% (SD = 17%). Thirteen (35%) PHUs manifested constant returns to scale, 21 (57%) decreasing returns to scale, and 3 (8%) increasing returns to scale.

## Discussion

The findings of this study reveal that more than half of the PHUs are operating at less than optimal levels of pure technical and scale efficiency. The performance of some of the PHUs in the sample is actually observed to be very low, and raises much concern for planners and policymakers. With the existing levels of inefficiency, the achievement of the health policy objectives and health-related global and regional targets such as the Millennium Development Goals (MDGs) and Abuja targets will be compromised. Hence, greater focus should be placed on efficient use of the existing resources.

The results obtained in Sierra Leone were similar to those obtained from the efficiency analysis of Kenyan health centres [17]. A study found 56% health centres in Kenya to be technically inefficient, with an average TE score of 65%. The average scale efficiency score among inefficient PHUs was 72% in Sierra Leone and 70% in Kenya. Seventy per cent of primary health care clinics in Kwazulu-Natal province in South Africa were found to be technically inefficient and 84% scale inefficient [18].

Table 6 shows the total output increases needed to make inefficient public PHUs efficient. In order to become efficient, the 22 inefficient PHUs combined would need to increase their current output levels by 57% more antenatal and postnatal care visits, 50% more deliveries, 85% more nutrition/growth monitoring visits, 45% more family planning visits, 40% more children and pregnant women who are fully immunized and 36% more health education sessions. This potential of providing more preventive health services to those currently without access, at no extra cost, would be of great public health importance in a poor country like Sierra Leone where large numbers of women do not have access to contraceptives, antenatal care and trained attendants during childbirth; where a large percentage of children are underweight, stunted and wasted; and where a large proportion of children do not have access to the Expanded Programme on Immunization (EPI) that targets diphtheria, tetanus, whooping cough, polio, tuberculosis and measles (Table 3). Also, over 50% of the population in the country does not have access to safe water, sanitation facilities and health care services. Thus, it is irrational, immoral and unethical to deny needy people access to essential health services through inefficiencies.

**Table 6:** Total output increases needed to make inefficient public PHUs efficient

**Outputs**	**Radial movement (A)**	**Slack movement (B)**	**Total value (A+B=C)**
Antenatal plus postnatal care visits	5 819	8 509	14 327
Number of deliveries	1 328	1 095	2 423
Nutrition/growth monitoring visits	5 903	19 398	25 301
Family planning visits	835	502	1 338
Number of children under the age of 5 years and pregnant women fully immunized	8 430	4 945	13 375
Health education sessions	1 964	744	2 708

The predominant form of scale inefficiency is decreasing returns to scale, which is also known as diseconomies of scale. A PHU operating at decreasing returns to scale has an inefficiently large size. A percentage increase in all inputs is followed by less than a percentage change in outputs. To improve the efficiency of the inefficiently large PHUs, there is a need to have more health units of a relatively smaller size.

Judging from the various statements contained in the national health policy and plan, and the health sector reforms that the Ministry of Health has been implementing; there is clearly a willingness to optimise the use of the scarce health resources.

While the scope for staff reduction in Pujehun District of Sierra Leone was almost non-existent as revealed by this study, there was certainly scope for providing essential public PHUs to a significantly larger number of people than the health units were actually providing. This could be achieved through a conscious pursuit of health promotion strategies [21] to create or induce demand for essential preventive public health services that were being under-utilized. Examples of such demand-inducing strategies might include:

(i) *Health information*: Improve people's ability to access health information to increase their capacity to make informed choices concerning their health-related behaviours, e.g. availing at community level information on the benefits of antenatal care, family planning, use of condoms to prevent HIV infection or transmission, immunization, safe water, hygienic sanitation facilities, abstinence from use of addictive substances (e.g. tobacco, alcohol and illicit drugs), physical activity and healthy diet. This strategy was important for primary prevention, which aimed at keeping a disease from ever developing or a trauma from ever occurring [22].

(ii) *Health education*: Communicating information concerning the underlying social, economic and environmental conditions impacting on health as well as individual risk factors and risk behaviours and use of the health system. In addition, health education was meant to foster motivation, skills and confidence among communities to take action to improve their health [21].

(iii) *Screening and individual risk assessment*: Identifying and assisting individuals at special risk to seek secondary prevention, which involve early detection and early intervention against disease before it developed fully, e.g. cervical cancer screening (pap smears) to identify pre-malignant cell changes [22], screening for intestinal nematode infections, ascariasis, *trichuriasis*, hookworm disease or tropical diseases (e.g. trypanosomiasis, schistosomiasis, lymphatic filariasis and onchocerciasis).

(iv) *Social marketing*: Attempting to influence communities living in the vicinity of health units on how to think and behave (with respect to utilization of preventive health services) by using marketing techniques [21]. The object of social marketing would be to cultivate positive attitudes, values and behaviours towards participation in disease prevention services.

The findings indicate that the amount of outputs could be increased tremendously without increasing the quantity of inputs used. As can be seen from Table 6, each of the outputs exhibits a tremendous increase – more than 50% in some cases. This includes both radial and slack movements. Radial movements indicate the proportional increase in outputs, that is, an increase without changing the mix of the outputs. The slack movements, which arise because of the sections of the piece-wise linear frontier that run parallel to the axes are also reported in order to give an accurate indication of the technical efficiency of the health centres. It should, however, be noted that sometimes slacks are treated as issues of allocative efficiency and therefore the focus is on the radial efficiency score. Thus, with the potential increase in outputs from the current sample of health centres, it is possible for the health system to significantly increase coverage by the different health interventions and contribute to the achievement of the various national and global health targets.

The extent to which the PHUs can increase their outputs depends on whether the health workers contract renewal and remunerations (especially annual increments) are linked to their performance. Currently, the health workers are paid salaries, which are not linked to performance. Efforts to improve health facility efficiency will need to be undertaken in tandem with reforms in health workers terms of employment. Such reforms are contemplated within the on-going public sector reforms, which are being supported by bilateral and multilateral development partners.

### Limitations of the study

Our study had some limitations. Firstly, in this study we used total number of health education sessions conducted through home visits, public meetings, school lectures and outpatient department as a proxy for health promotion. By so doing we may have underestimated the health promotion work that is done by health centre staff within communities, e.g. public health inspection of commercial food outlets, coaching of communities on personal hygiene, advise to communities on the protection of water sources and construction of vented improved pit latrines (in rural areas and shanties), etc.

Secondly, the inputs and outputs data were collected for only one time period; thus, it was not possible to determine whether the health sector reforms had any impact on the efficiency of PHUs. Thirdly, data on drug expenditure at many PHUs were missing; as a result we were forced to drop the variable from the analysis, which may result in shifting the frontier because of outlier figures. Fourthly, we did not manage to collect information on input prices, and thus, we could not estimate the allocative efficiency of the PHUs. Fifthly, given that the study were conducted in only one district, it would not be advisable to generalize the findings to the whole country. Thus, it is recommended that the study should be replicated in the remaining twelve districts. Lastly, DEA has been criticized for attributing any deviation from the estimated frontier to inefficiency since it is deterministic or non-stochastic [23,24]. In other words, it does not capture random noise, e.g. epidemics, civil war and natural and technological disasters.

To increase the relevance of the study for management purposes, it would have been useful to undertake a second stage analysis of the factors influencing inefficiency using a Tobit – censored dependent variable model – regression analysis. However, because of the absence of good quality data on the factors often hypothesized to influence inefficiency it was not possible to undertake the analysis.

### Implications for further applications of DEA in sub-Saharan Africa

A national health system performs the functions of stewardship (oversight), health financing (revenue collection, pooling of resources and sharing of financial risk, purchasing of health services), creating resources/inputs (including human resources for health) for producing health, and providing health services with a view to improving responsiveness to people's non-medical expectations, ensuring fair financial contribution to health systems and ultimately improving health (the three being goals of health system) [6].

The World Health Report 2000 ranked the 191 Member States on the basis of their overall health system goal performance. Table 7 provides the ranking of the 46 countries in the WHO African Region: 3 countries were ranked between 83 and 99; 9 countries were ranked between 118 and 147; and the remaining countries were ranked between 151 and 191. The Sierra Leone health system performed the worst.

**Table 7:** Ranking of WHO African Region countries on the basis of overall health system goal attainment (out of 191 WHO Member States in 2000)

**Member State**	**Ranking**	**Member State**	**Rank**
Seychelles	83	Swaziland	164
Mauritius	90	Namibia	165
Algeria	99	Madagascar	167
Senegal	118	Botswana	168
Cape Verde	126	Mauritania	169
Comoros	137	Rwanda	171
Sao Tome and Principe	138	Guinea	172
Ghana	139	Lesotho	173
Kenya	142	Zambia	174
Benin	143	Eritrea	176
Gabon	147	Chad	177
Zimbabwe	147	Mali	178
South Africa	151	Democratic Republic of Congo	179
Equatorial Guinea	152	Guinea-Bissau	180
Gambia	153	Angola	181
Congo	155	Malawi	182
Togo	156	Nigeria	184
Cote d'Ivoire	157	Mozambique	185
United Republic of Tanzania	158	Ethiopia	186
Burkina Faso	159	Liberia	187
Burundi	161	Niger	188
Uganda	162	Central African Republic	190
Cameroon	163	Sierra Leone	191

Source: WHO [6].

After the publication of these macro-performance results, countries in the Region have been asking what they can do to improve the performance of their health systems, or even performance of their individual hospitals and health centres which absorb over 80% of recurrent and capital/development budgets of the Ministries of Health. The starting point in addressing the poor health system performance, is measuring which decision-making units (tertiary hospitals, provincial hospitals, health centres, clinics/health posts, programmes) (DMU) of the present system are operating efficiently. These measurements can help identify: efficient DMUs, whose practise can be emulated by the inefficient DMUs; inefficient DMUs, whose performance need to be improved; the inputs that are being wasted and the magnitude of waste; and the output increases needed to make inefficient DMUs efficient. This kind of evidence would empower health policy makers and managers to develop concrete strategies for boosting efficiency of DMUs. As demonstrated in the current study, DEA is a useful tool/approach for analysing the efficiency of complex DMUs (e.g. hospitals, health centres) that employ multiple inputs to produce multiple outputs, with a view to generating the evidence mentioned above.

Efficiency improvement is a major strategy for mobilizing more domestic resources for the massive expansion in the coverage of health interventions envisaged in the Millennium Development Goals. Thus, while striving to mobilize more domestic and external resources, it is important to ensure that the available resources are optimally used, i.e. ensure that it is not possible by reallocation of available resources to make one person's health status better off without making another person's health status worse off (this situation is called by economists Pareto-optimality). If it is possible through reallocation of resources to improve at least one person's health status without reducing health status of another person, then there is waste within the health system, health facility or programme.

Therefore, we strongly recommend that every country in the Region should institutionalise health facility efficiency monitoring at the Ministry of Health headquarter (MoH/HQ) and at each health district headquarter. In the process of institutionalisation, there will be need to: (i) familiarize the policy makers (ministers, permanent secretaries, directors of medical services), managers (MoH/HQ departmental heads, provincial medical officers of health, district medical officers of health, hospital superintendents) and economists (and planners) at the Ministry of Health with the concepts of technical efficiency, allocative efficiency and total factor productivity; (ii) acquire computers (where they do not exist) and software's (parametric and non-parametric) for estimating efficiency; (iii) organize hands-on training for MoH economists and planners (and where possible provincial and district health managers) in the use of the efficiency measurement software's; (iv) adapt the available efficiency data collection questionnaires/instruments; (v) undertake a pilot study among a few different level health facilities and revise the data collection instruments accordingly; (vi) make the data collection instruments part of the national health information systems; (vii) decide on the frequency of reporting of the inputs (quantities and prices) and outputs by those in charge of health facilities; (viii) the analysis could be undertaken with at the district level (with MoH/HQ support) with a view to identifying causes of inefficiencies, developing strategies for improving efficiency and implementing them; (ix) establish efficiency database at MoH/HQ and at each health district headquarters.

## Conclusion

DEA has been fruitfully used in many countries in Asia [25,26] and Europe [27-32] and in the United States [33-37] to shed light on the efficiency of health facilities and programmes. The current study adds to this literature. The study has revealed the prevalence of high levels of combined pure technical and scale inefficiencies. In a country with very low levels of per capita expenditure on health (US$7) and very limited access to health services, the current levels of inefficiency would seriously impede the government's initiatives to increase the population's access to quality health care services. Furthermore, progress towards the achievement of the cherished health policy objectives, and global and regional health targets would be seriously hampered.

It is therefore recommended that the causes of the inefficiencies be unpacked and necessary efficiency measures be instituted to augment the government's efforts to address the health care access issues in the country. To estimate the level of efficiency savings in the overall health system, it is also advisable to undertake a similar study in all types of health facilities in the country.

In any efficiency analysis studies to be conducted in Sierra Leone in the future, more emphasis should be laid on collecting information on the quantities of all the main outputs and inputs (including drugs) and the average or median prices per unit of each input, from all public and private health facilities (health centres and hospitals), to facilitate measurement of total economic efficiencies (i.e. technical plus allocative efficiencies). Furthermore, in order to aid monitoring and evaluation of the effects of different health care reforms [38] on the efficiency of individual health care facilities over time through the *Malmquist *Productivity Index analysis [16,39], it would be necessary to collect data for a year (or more) before the introduction of specific reforms, and for subsequent years. The Malmquist Productivity Index helps to measure explicitly total factor productivity. It decomposes productivity growth into efficiency change and technical change. The former component is considered to be evidence of catching up to the efficiency frontier, while the latter component is considered to be evidence of innovation [39].

## Competing interests

The author(s) declare that they have no competing interests.

## Authors' contributions

AR supervised data collection and participated in writing of sections of the manuscript. JMK analysed the data, participated in developing the conceptual framework, literature review and writing various sections of the manuscript. EZA participated in developing the conceptual framework and writing various sections of the manuscript. SB participated in the literature review and writing sections of the manuscript. DGK and LHKM contributed to various sections of the manuscript and wrote aspects of the discussion on health promotion strategies. CK coordinated the entire study and wrote the subsections entitled "overview of Sierra Leone health care delivery system" and "data".

## Pre-publication history

The pre-publication history for this paper can be accessed here:


